# Strongly Convex Divergences

**DOI:** 10.3390/e22111327

**Published:** 2020-11-21

**Authors:** James Melbourne

**Affiliations:** Department of Electrical and Computer Engineering, University of Minnesota-Twin Cities, Minneapolis, MN 55455, USA; melbo013@umn.edu

**Keywords:** information measures, f-divergence, hypothesis testing, total variation, skew-divergence, convexity, Pinsker’s inequality, Bayes risk, Jensen–Shannon divergence

## Abstract

We consider a sub-class of the *f*-divergences satisfying a stronger convexity property, which we refer to as strongly convex, or κ-convex divergences. We derive new and old relationships, based on convexity arguments, between popular *f*-divergences.

## 1. Introduction

The concept of an *f*-divergence, introduced independently by Ali-Silvey [1], Morimoto [2], and Csisizár [3], unifies several important information measures between probability distributions, as integrals of a convex function *f*, composed with the Radon–Nikodym of the two probability distributions. (An additional assumption can be made that *f* is strictly convex at 1, to ensure that $D_{f}\left(\mu ||\nu \right)>0$ for μ≠ν. This obviously holds for any $f^{\prime \prime }\left(1\right)>0$, and can hold for some *f*-divergences without classical derivatives at 0, for instance the total variation is strictly convex at 1. An example of an *f*-divergence not strictly convex is provided by the so-called “hockey-stick” divergence, where $f\left(x\right)={\left(x-\gamma \right)}_{+}$, see [4,5,6].) For a convex function f:(0,∞)→R such that f(1)=0, and measures *P* and *Q* such that P≪Q, the *f*-divergence from *P* to *Q* is given by $D_{f}\left(P||Q\right):=\int f\left(\frac{dP}{dQ}\right)dQ.$ The canonical example of an *f*-divergence, realized by taking f(x)=xlogx, is the relative entropy (often called the KL-divergence), which we denote with the subscript *f* omitted. *f*-divergences inherit many properties enjoyed by this special case; non-negativity, joint convexity of arguments, and a data processing inequality. Other important examples include the total variation, the $\chi^{2}$-divergence, and the squared Hellinger distance. The reader is directed to Chapter 6 and 7 of [7] for more background.

We are interested in how stronger convexity properties of *f* give improvements of classical *f*-divergence inequalities. More explicitly, we consider consequences of *f* being κ-convex, in the sense that the map $x\mapsto f\left(x\right)-\kappa x^{2}/2$ is convex. This is in part inspired by the work of Sason [8], who demonstrated that divergences that are κ-convex satisfy “stronger than $\chi^{2}$” data-processing inequalities.

Perhaps the most well known example of an *f*-divergence inequality is Pinsker’s inequality, which bounds the square of the total variation above by a constant multiple of the relative entropy. That is for probability measures *P* and *Q*, ${\left|P-Q\right|}_{TV}^{2}\leq c\;D\left(P||Q\right)$. The optimal constant is achieved for Bernoulli measures, and under our conventions for total variation, c=1/2loge. Many extensions and sharpenings of Pinsker’s inequality exist (for examples, see [9,10,11]). Building on the work of Guntuboyina [9] and Topsøe [11], we achieve a further sharpening of Pinsker’s inequality in Theorem 9.

Aside from the total variation, most divergences of interest have stronger than affine convexity, at least when *f* is restricted to a sub-interval of the real line. This observation is especially relevant to the situation in which one wishes to study $D_{f}\left(P||Q\right)$ in the existence of a bounded Radon–Nikodym derivative $\frac{dP}{dQ}\in \left(a,b\right)⊊\left(0,\infty \right)$. One naturally obtains such bounds for skew divergences. That is divergences of the form $\left(P,Q\right)\mapsto D_{f}\left(\left(1-t\right)P+tQ||\left(1-s\right)P+sQ\right)$ for t,s∈[0,1], as in this case, $\frac{(1-t)P+tQ}{(1-s)P+sQ}\leq \max\left\{\frac{1-t}{1-s},\frac{t}{s}\right\}$. Important examples of skew-divergences include the skew divergence [12] based on the relative entropy and the Vincze–Le Cam divergence [13,14], called the triangular discrimination in [11] and its generalization due to Györfi and Vajda [15] based on the $\chi^{2}$-divergence. The Jensen–Shannon divergence [16] and its recent generalization [17] give examples of *f*-divergences realized as linear combinations of skewed divergences.

Let us outline the paper. In Section 2, we derive elementary results of κ-convex divergences and give a table of examples of κ-convex divergences. We demonstrate that κ-convex divergences can be lower bounded by the $\chi^{2}$-divergence, and that the joint convexity of the map $\left(P,Q\right)\mapsto D_{f}\left(P||Q\right)$ can be sharpened under κ-convexity conditions on *f*. As a consequence, we obtain bounds between the mean square total variation distance of a set of distributions from its barycenter, and the average *f*-divergence from the set to the barycenter.

In Section 3, we investigate general skewing of *f*-divergences. In particular, we introduce the skew-symmetrization of an *f*-divergence, which recovers the Jensen–Shannon divergence and the Vincze–Le Cam divergences as special cases. We also show that a scaling of the Vincze–Le Cam divergence is minimal among skew-symmetrizations of κ-convex divergences on (0,2). We then consider linear combinations of skew divergences and show that a generalized Vincze–Le Cam divergence (based on skewing the $\chi^{2}$-divergence) can be upper bounded by the generalized Jensen–Shannon divergence introduced recently by Nielsen [17] (based on skewing the relative entropy), reversing the classical convexity bounds $D\left(P||Q\right)\leq \log(1+\chi^{2}\left(P||Q)\right)\leq \log e\;\chi^{2}\left(P||Q\right)$. We also derive upper and lower total variation bounds for Nielsen’s generalized Jensen–Shannon divergence.

In Section 4, we consider a family of densities $\left\{p_{i}\right\}$ weighted by $\lambda_{i}$, and a density *q*. We use the Bayes estimator $T\left(x\right)=\arg\max_{i}\lambda_{i}p_{i}\left(x\right)$ to derive a convex decomposition of the barycenter $p=\sum_{i}\lambda_{i}p_{i}$ and of *q*, each into two auxiliary densities. (Recall, a Bayes estimator is one that minimizes the expected value of a loss function. By the assumptions of our model, that $P\left(\theta =i\right)=\lambda_{i}$, and $P\left(X\in A|\theta =i\right)=\int_{A}p_{i}\left(x\right)dx$, we have $E\ell \left(\theta ,\hat{\theta }\right)=1-\int \lambda_{\hat{\theta }\left(x\right)}p_{\hat{\theta }\left(x\right)}\left(x\right)dx$ for the loss function $\ell \left(i,j\right)=1-\delta_{i}\left(j\right)$ and any estimator $\hat{\theta }$. It follows that $E\ell \left(\theta ,\hat{\theta }\right)\geq E\ell \left(\theta ,T\right)$ by $\lambda_{\hat{\theta }\left(x\right)}p_{\hat{\theta }\left(x\right)}\left(x\right)\leq \lambda_{T(x)}p_{T(x)}\left(x\right)$. Thus, *T* is a Bayes estimator associated to *ℓ*. ) We use this decomposition to sharpen, for κ-convex divergences, an elegant theorem of Guntuboyina [9] that generalizes Fano and Pinsker’s inequality to *f*-divergences. We then demonstrate explicitly, using an argument of Topsøe, how our sharpening of Guntuboyina’s inequality gives a new sharpening of Pinsker’s inequality in terms of the convex decomposition induced by the Bayes estimator.

### Notation

Throughout, *f* denotes a convex function f:(0,∞)→R∪{∞}, such that f(1)=0. For a convex function defined on (0,∞), we define $f\left(0\right):=\lim_{x\to 0}f\left(x\right)$. We denote by $f^{*}$, the convex function $f^{*}:\left(0,\infty \right)\to R\cup \left\{\infty \right\}$ defined by $f^{*}\left(x\right)=xf\left(x^{-1}\right)$. We consider Borel probability measures *P* and *Q* on a Polish space X and define the *f*-divergence from *P* to *Q*, via densities *p* for *P* and *q* for *Q* with respect to a common reference measure μ as

(1) $$\begin{array}{cc} D_{f}\left(p||q\right) & =\int_{X}f\left(\frac{p}{q}\right)qd\mu \\ \; & =\int_{\left\{pq>0\right\}}qf\left(\frac{p}{q}\right)d\mu +f\left(0\right)Q\left(\left\{p=0\right\}\right)+f^{*}\left(0\right)P\left(\left\{q=0\right\}\right). \end{array}$$

We note that this representation is independent of μ, and such a reference measure always exists, take μ=P+Q for example.

For t,s∈[0,1], define the binary *f*-divergence

(2) $$D_{f}\left(t||s\right):=sf\left(\frac{t}{s}\right)+\left(1-s\right)f\left(\frac{1-t}{1-s}\right)$$

with the conventions, $f\left(0\right)=\lim_{t\to 0^{+}}f\left(t\right)$, 0f(0/0)=0, and $0f\left(a/0\right)=a\lim_{t\to \infty }f\left(t\right)/t$. For a random variable *X* and a set *A*, we denote the probability that *X* takes a value in *A* by P(X∈A), the expectation of the random variable by EX, and the variance by ${\mathrm{Var}\left(X\right):=E|X-EX|}^{2}$. For a probability measure μ satisfying μ(A)=P(X∈A) for all Borel *A*, we write X∼μ, and, when there exists a probability density function such that $P\left(X\in A\right)=\int_{A}f\left(x\right)d\gamma \left(x\right)$ for a reference measure γ, we write X∼f. For a probability measure μ on X, and an $L^{2}$ function f:X→R, we denote ${\mathrm{Var}}_{\mu }\left(f\right):=\mathrm{Var}\left(f\left(X\right)\right)$ for X∼μ.

## 2. Strongly Convex Divergences

**Definition** **1.** *A R∪{∞}-valued function f on a convex set K⊆R is κ-convex when x,y∈K and t∈[0,1] implies*

(3) $$f\left(\left(1-t\right)x+ty\right)\leq \left(1-t\right)f\left(x\right)+tf\left(y\right)-\kappa t\left(1-t\right){\left(x-y\right)}^{2}/2.$$

For example, when *f* is twice differentiable, (3) is equivalent to $f^{\prime \prime }\left(x\right)\geq \kappa$ for x∈K. Note that the case κ=0 is just usual convexity.

**Proposition** **1.** *For f:K→R∪{∞} and κ∈[0,∞), the following are equivalent:*

1. *f is κ-convex.*
2. *The function $f-\kappa {\left(t-a\right)}^{2}/2$ is convex for any a∈R.*
3. *The right handed derivative, defined as $f_{+}^{\prime }\left(t\right):=\lim_{h↓0}\frac{f(t+h)-f(t)}{h}$ satisfies,*
  $$f_{+}^{\prime }\left(t\right)\geq f_{+}^{\prime }\left(s\right)+\kappa \left(t-s\right)$$
*for t≥s.*

**Proof.**  Observe that it is enough to prove the result when κ=0, where the proposition is reduced to the classical result for convex functions. □

**Definition** **2.** *An f-divergence $D_{f}$ is κ-convex on an interval K for κ≥0 when the function f is κ-convex on K.*

Table 1 lists some κ-convex *f*-divergences of interest to this article.

Observe that we have taken the normalization convention on the total variation (the total variation for a signed measure μ on a space *X* can be defined through the Hahn-Jordan decomposition of the measure into non-negative measures $\mu^{+}$ and $\mu^{-}$ such that $\mu =\mu^{+}-\mu^{-}$, as $∥\mu ∥=\mu^{+}\left(X\right)+\mu^{-}\left(X\right)$ (see [18]); in our notation, ${\left|\mu \right|}_{TV}=∥\mu ∥/2$) which we denote by ${\left|P-Q\right|}_{TV}$, such that ${\left|P-Q\right|}_{TV}=\sup_{A}\left|P\left(A\right)-Q\left(A\right)\right|\leq 1$. In addition, note that the α-divergence interpolates Pearson’s $\chi^{2}$-divergence when α=3, one half Neyman’s $\chi^{2}$-divergence when α=−3, the squared Hellinger divergence when α=0, and has limiting cases, the relative entropy when α=1 and the reverse relative entropy when α=−1. If *f* is κ-convex on [a,b], then recalling its dual divergence $f^{*}\left(x\right):=xf\left(x^{-1}\right)$ is $\kappa a^{3}$-convex on $\left[\frac{1}{b},\frac{1}{a}\right]$. Recall that $f^{*}$ satisfies the equality $D_{f^{*}}\left(P||Q\right)=D_{f}\left(Q||P\right)$. For brevity, we use $\chi^{2}$-divergence to refer to the Pearson $\chi^{2}$-divergence, and we articulate Neyman’s $\chi^{2}$ explicitly when necessary.

The next lemma is a restatement of Jensen’s inequality.

**Lemma** **1.** *If f is κ-convex on the range of X,*

$$Ef\left(X\right)\geq f\left(E\left(X\right)\right)+\frac{\kappa }{2}\mathrm{Var}\left(X\right).$$

**Proof.**  Apply Jensen’s inequality to $f\left(x\right)-\kappa x^{2}/2$. □

For a convex function *f* such that f(1)=0 and c∈R, the function $\tilde{f}\left(t\right)=f\left(t\right)+c\left(t-1\right)$ remains a convex function, and what is more satisfies

$$D_{f}\left(P||Q\right)=D_{\tilde{f}}\left(P||Q\right)$$

since ∫c(p/q−1)qdμ=0.

**Definition** **3** ($\chi^{2}$-divergence)**.** *For $f\left(t\right)={\left(t-1\right)}^{2}$, we write*

$$\chi^{2}\left(P||Q\right):=D_{f}\left(P||Q\right).$$

We pursue a generalization of the following bound on the total variation by the $\chi^{2}$-divergence [19,20,21].

**Theorem** **1** ([19,20,21])**.** *For measures P and Q,*

(4) $$\begin{array}{c} {\left|P-Q\right|}_{TV}^{2}\leq \frac{\chi^{2}\left(P||Q\right)}{2}. \end{array}$$

We mention the work of Harremos and Vadja [20], in which it is shown, through a characterization of the extreme points of the joint range associated to a pair of *f*-divergences (valid in general), that the inequality characterizes the “joint range”, that is, the range of the function $\left(P,Q\right)\mapsto {\left(|P-Q\right|}_{TV},\chi^{2}\left(P||Q)\right)$. We use the following lemma, which shows that every strongly convex divergence can be lower bounded, up to its convexity constant κ>0, by the $\chi^{2}$-divergence,

**Lemma** **2.** *For a κ-convex f,*

$$D_{f}\left(P||Q\right)\geq \frac{\kappa }{2}\chi^{2}\left(P||Q\right).$$

**Proof.**  Define a $\tilde{f}\left(t\right)=f\left(t\right)-f_{+}^{\prime }\left(1\right)\left(t-1\right)$ and note that $\tilde{f}$ defines the same κ-convex divergence as *f*. Thus, we may assume without loss of generality that $f_{+}^{\prime }$ is uniquely zero when t=1. Since *f* is κ-convex $\phi :t\mapsto f\left(t\right)-\kappa {\left(t-1\right)}^{2}/2$ is convex, and, by $f_{+}^{\prime }\left(1\right)=0$, $\phi_{+}^{\prime }\left(1\right)=0$ as well. Thus, ϕ takes its minimum when t=1 and hence ϕ≥0 so that $f\left(t\right)\geq \kappa {\left(t-1\right)}^{2}/2$. Computing,

$$\begin{array}{cc} D_{f}\left(P||Q\right) & =\int f\left(\frac{dP}{dQ}\right)dQ\\ \; & \geq \frac{\kappa }{2}\int {\left(\frac{dP}{dQ}-1\right)}^{2}dQ\\ \; & =\frac{\kappa }{2}\chi^{2}\left(P||Q\right). \end{array}$$

 □

Based on a Taylor series expansion of *f* about 1, Nielsen and Nock ([22], [Corollary 1]) gave the estimate

(5) $$\begin{array}{c} D_{f}\left(P||Q\right)\approx \frac{f^{\prime \prime }\left(1\right)}{2}\chi^{2}\left(P||Q\right) \end{array}$$

for divergences with a non-zero second derivative and *P* close to *Q*. Lemma 2 complements this estimate with a lower bound, when *f* is κ-concave. In particular, if $f^{\prime \prime }\left(1\right)=\kappa$, it shows that the approximation in (5) is an underestimate.

**Theorem** **2.** *For measures P and Q, and a κ convex divergence $D_{f}$,*

(6) $$\begin{array}{c} {\left|P-Q\right|}_{TV}^{2}\leq \frac{D_{f}\left(P||Q\right)}{\kappa }. \end{array}$$

**Proof.**  By Lemma 2 and then Theorem 1,

(7) $$\begin{array}{c} \frac{D_{f}\left(P||Q\right)}{\kappa }\geq \frac{\chi^{2}\left(P||Q\right)}{2}\geq {\left|P-Q\right|}_{TV}. \end{array}$$

 □

The proof of Lemma 2 uses a pointwise inequality between convex functions to derive an inequality between their respective divergences. This simple technique was shown to have useful implications by Sason and Verdu in [6], where it appears as Theorem 1 and is used to give sharp comparisons in several *f*-divergence inequalities.

**Theorem** **3** (Sason–Verdu [6])**.** *For divergences defined by g and f with cf(t)≥g(t) for all t, then*

$$D_{g}\left(P||Q\right)\leq cD_{f}\left(P||Q\right).$$

*Moreover, if $f^{\prime }\left(1\right)=g^{\prime }\left(1\right)=0$, then*

$$\underset{P\neq Q}{\sup}\frac{D_{g}\left(P||Q\right)}{D_{f}\left(P||Q\right)}=\underset{t\neq 1}{\sup}\frac{g(t)}{f(t)}.$$

**Corollary** **1.** *For a smooth κ-convex divergence f, the inequality*

(8) $$D_{f}\left(P||Q\right)\geq \frac{\kappa }{2}\chi^{2}\left(P||Q\right)$$

*is sharp multiplicatively in the sense that*

(9) $$\underset{P\neq Q}{\inf}\frac{D_{f}\left(P||Q\right)}{\chi^{2}\left(P||Q\right)}=\frac{\kappa }{2}.$$

*if $f^{\prime \prime }\left(1\right)=\kappa$.*

In information geometry, a standard *f*-divergence is defined as an *f*-divergence satisfying the normalization $f\left(1\right)=f^{\prime }\left(1\right)=0,f^{\prime \prime }\left(1\right)=1$ (see [23]). Thus, Corollary 1 shows that $\frac{1}{2}\chi^{2}$ provides a sharp lower bound on every standard *f*-divergence that is 1-convex. In particular, the lower bound in Lemma 2 complimenting the estimate (5) is shown to be sharp.

**Proof.**  Without loss of generality, we assume that $f^{\prime }\left(1\right)=0$. If $f^{\prime \prime }\left(1\right)=\kappa +2\varepsilon$ for some ε>0, then taking $g\left(t\right)={\left(t-1\right)}^{2}$ and applying Theorem 3 and Lemma 2

(10) $$\underset{P\neq Q}{\sup}\frac{D_{g}\left(P||Q\right)}{D_{f}\left(P||Q\right)}=\underset{t\neq 1}{\sup}\frac{g(t)}{f(t)}\leq \frac{2}{\kappa }.$$

Observe that, after two applications of L’Hospital,

$$\lim_{\varepsilon \to 0}\frac{g(1+\varepsilon )}{f(1+\varepsilon )}=\lim_{\varepsilon \to 0}\frac{g^{\prime }\left(1+\varepsilon \right)}{f^{\prime }\left(1+\varepsilon \right)}=\frac{g^{\prime \prime }\left(1\right)}{f^{\prime \prime }\left(1\right)}=\frac{2}{\kappa }\leq \underset{t\neq 1}{\sup}\frac{g(t)}{f(t)}.$$

Thus, (9) follows. □

**Proposition** **2.** *When $D_{f}$ is an f divergence such that f is κ-convex on [a,b] and that $P_{\theta }$ and $Q_{\theta }$ are probability measures indexed by a set *Θ* such that $a\leq \frac{dP_{\theta }}{dQ_{\theta }}\left(x\right)\leq b$, holds for all θ and $P:=\int_{\Theta }P_{\theta }d\mu \left(\theta \right)$ and $Q:=\int_{\Theta }Q_{\theta }d\mu \left(\theta \right)$ for a probability measure μ on *Θ*, then*

(11) $$D_{f}\left(P||Q\right)\leq \int_{\Theta }D_{f}(P_{\theta }\left|\right|Q_{\theta })d\mu \left(\theta \right)-\frac{\kappa }{2}\int_{\Theta }\int_{X}{\left(\frac{dP_{\theta }}{dQ_{\theta }}-\frac{dP}{dQ}\right)}^{2}dQd\mu ,$$

*In particular, when $Q_{\theta }=Q$ for all θ*

(12) $$\begin{array}{c} D_{f}(P||Q)\\ \;\;\leq \int_{\Theta }D_{f}(P_{\theta }||Q)d\mu \left(\theta \right)-\frac{\kappa }{2}\int_{\Theta }\int_{X}{\left(\frac{dP_{\theta }}{dQ}-\frac{dP}{dQ}\right)}^{2}dQd\mu \left(\theta \right)\\ \;\;\leq \int_{\Theta }D_{f}(P_{\theta }||Q)d\mu \left(\theta \right)-\kappa \int_{\Theta }{\left|P_{\theta }-P\right|}_{TV}^{2}d\mu \left(\theta \right) \end{array}$$

**Proof.**  Let dθ denote a reference measure dominating μ so that dμ=φ(θ)dθ then write $\nu_{\theta }=\nu \left(\theta ,x\right)=\frac{dQ_{\theta }}{dQ}\left(x\right)\varphi \left(\theta \right)$.

(13) $$\begin{array}{cc} D_{f}\left(P||Q\right) & =\int_{X}f\left(\frac{dP}{dQ}\right)dQ\\ \; & =\int_{X}f\left(\int_{\Theta }\frac{dP_{\theta }}{dQ}d\mu \left(\theta \right)\right)dQ\\ \; & =\int_{X}f\left(\int_{\Theta }\frac{dP_{\theta }}{dQ_{\theta }}\nu \left(\theta ,x\right)d\theta \right)dQ \end{array}$$

By Jensen’s inequality, as in Lemma 1

$$\begin{array}{cc} f\left(\int_{\Theta }\frac{dP_{\theta }}{dQ_{\theta }}\nu_{\theta }d\theta \right)\leq \int_{\theta }f & \left(\frac{dP_{\theta }}{dQ_{\theta }}\right)\nu_{\theta }d\theta -\frac{\kappa }{2}\int_{\Theta }{\left(\frac{dP_{\theta }}{dQ_{\theta }}-\int_{\Theta }\frac{dP_{\theta }}{dQ_{\theta }}\nu_{\theta }d\theta \right)}^{2}\nu_{\theta }d\theta \end{array}$$

Integrating this inequality gives

(14) $$D_{f}\left(P||Q\right)\leq \int_{X}\left(\int_{\theta }f\left(\frac{dP_{\theta }}{dQ_{\theta }}\right)\nu_{\theta }d\theta -\frac{\kappa }{2}\int_{\Theta }{\left(\frac{dP_{\theta }}{dQ_{\theta }}-\int_{\Theta }\frac{dP_{\theta }}{dQ_{\theta }}\nu_{\theta }d\theta \right)}^{2}\nu_{\theta }d\theta \right)dQ$$

Note that

$$\int_{X}\int_{\Theta }{\left(\frac{dP_{\theta }}{dQ_{\theta }}dQ-\int_{\Theta }\frac{dP_{\theta }}{dQ_{\theta_{0}}}\nu_{\theta_{0}}d\theta_{0}\right)}^{2}\nu_{\theta }d\theta dQ=\int_{\Theta }\int_{X}{\left(\frac{dP_{\theta }}{dQ_{\theta }}-\frac{dP}{dQ}\right)}^{2}dQd\mu ,$$

and

(15) $$\begin{array}{cc} \int_{X}\int_{\Theta }f\left(\frac{dP_{\theta }}{dQ_{\theta }}\right)\nu \left(\theta ,x\right)d\theta dQ & =\int_{\Theta }\int_{X}f\left(\frac{dP_{\theta }}{dQ_{\theta }}\right)\nu \left(\theta ,x\right)dQd\theta \\ \; & =\int_{\Theta }\int_{X}f\left(\frac{dP_{\theta }}{dQ_{\theta }}\right)dQ_{\theta }d\mu \left(\theta \right)\\ \; & =\int_{\Theta }D(P_{\theta }\left|\right|Q_{\theta })d\mu \left(\theta \right) \end{array}$$

Inserting these equalities into (14) gives the result.

To obtain the total variation bound, one needs only to apply Jensen’s inequality,

(16) $$\begin{array}{cc} \int_{X}{\left(\frac{dP_{\theta }}{dQ}-\frac{dP}{dQ}\right)}^{2}dQ & \geq {\left(\int_{X}\left|\frac{dP_{\theta }}{dQ}-\frac{dP}{dQ}\right|dQ\right)}^{2}\\ \; & =|P_{\theta }{-P|}_{TV}^{2}. \end{array}$$

 □

Observe that, taking $Q=P=\int_{\Theta }P_{\theta }d\mu \left(\theta \right)$ in Proposition 2, one obtains a lower bound for the average *f*-divergence from the set of distribution to their barycenter, by the mean square total variation of the set of distributions to the barycenter,

(17) $$\kappa \int_{\Theta }|P_{\theta }{-P|}_{TV}^{2}d\mu \left(\theta \right)\leq \int_{\Theta }D_{f}(P_{\theta }||P)d\mu \left(\theta \right).$$

An alternative proof of this can be obtained by applying $|P_{\theta }{-P|}_{TV}^{2}\leq D_{f}(P_{\theta }\left||P\right)/\kappa$ from Theorem 2 pointwise.

The next result shows that, for *f* strongly convex, Pinsker type inequalities can never be reversed,

**Proposition** **3.** *Given f strongly convex and M>0, there exists P, Q measures such that*

(18) $$D_{f}\left(P||Q\right)\geq {M|P-Q|}_{TV}.$$

**Proof.**  By κ-convexity $\phi \left(t\right)=f\left(t\right)-\kappa t^{2}/2$ is a convex function. Thus, $\phi \left(t\right)\geq \phi \left(1\right)+\phi_{+}^{\prime }\left(1\right)\left(t-1\right)=\left(f_{+}^{\prime }\left(1\right)-\kappa \right)\left(t-1\right)$ and hence $\lim_{t\to \infty }\frac{f(t)}{t}\geq \lim_{t\to \infty }\kappa t/2+\left(f_{+}^{\prime }\left(1\right)-\kappa \right)\left(1-\frac{1}{t}\right)=\infty .$ Taking measures on the two points space P={1/2,1/2} and Q={1/2t,1−1/2t} gives $D_{f}\left(P||Q\right)\geq \frac{1}{2}\frac{f(t)}{t}$ which tends to infinity with t→∞, while ${\left|P-Q\right|}_{TV}\leq 1$. □

In fact, building on the work of Basu-Shioya-Park [24] and Vadja [25], Sason and Verdu proved [6] that, for any *f* divergence, $\sup_{P\neq Q}\frac{D_{f}\left(P||Q\right)}{{\left|P-Q\right|}_{TV}}=f\left(0\right)+f^{*}\left(0\right)$. Thus, an *f*-divergence can be bounded above by a constant multiple of a the total variation, if and only if $f\left(0\right)+f^{*}\left(0\right)<\infty$. From this perspective, Proposition 3 is simply the obvious fact that strongly convex functions have super linear (at least quadratic) growth at infinity.

## 3. Skew Divergences

If we denote Cvx(0,∞) to be quotient of the cone of convex functions *f* on (0,∞) such that f(1)=0 under the equivalence relation $f_{1}∼f_{2}$ when $f_{1}-f_{2}=c\left(x-1\right)$ for c∈R, then the map $f\mapsto D_{f}$ gives a linear isomorphism between Cvx(0,∞) and the space of all *f*-divergences. The mapping T:Cvx(0,∞)→Cvx(0,∞) defined by $Tf=f^{*}$, where we recall $f^{*}\left(t\right)=tf\left(t^{-1}\right)$, gives an involution of Cvx(0,∞). Indeed, $D_{Tf}\left(P||Q\right)=D_{f}\left(Q||P\right)$, so that $D_{T(T(f))}\left(P||Q\right)=D_{f}\left(P||Q\right)$. Mathematically, skew divergences give an interpolation of this involution as

$$\left(P,Q\right)\mapsto D_{f}\left(\left(1-t\right)P+tQ||\left(1-s\right)P+sQ\right)$$

gives $D_{f}\left(P||Q\right)$ by taking s=1 and t=0 or yields $D_{f^{*}}\left(P||Q\right)$ by taking s=0 and t=1.

Moreover, as mentioned in the Introduction, skewing imposes boundedness of the Radon–Nikodym derivative $\frac{dP}{dQ}$, which allows us to constrain the domain of *f*-divergences and leverage κ-convexity to obtain *f*-divergence inequalities in this section.

The following appears as Theorem III.1 in the preprint [26]. It states that skewing an *f*-divergence preserves its status as such. This guarantees that the generalized skew divergences of this section are indeed *f*-divergences. A proof is given in the Appendix A for the convenience of the reader.

**Theorem** **4** (Melbourne et al [26])**.** *For t,s∈[0,1] and a divergence $D_{f}$, then*

(19) $$S_{f}\left(P||Q\right):=D_{f}\left(\left(1-t\right)P+tQ||\left(1-s\right)P+sQ\right)$$

*is an f-divergence as well.*

**Definition** **4.** *For an f-divergence, its skew symmetrization,*

$$\Delta_{f}\left(P||Q\right):=\frac{1}{2}D_{f}\left(P||\frac{P+Q}{2}\right)+\frac{1}{2}D_{f}\left(Q||\frac{P+Q}{2}\right).$$

$\Delta_{f}$ is determined by the convex function

(20) $$x\mapsto \frac{1+x}{2}\left(f\left(\frac{2x}{1+x}\right)+f\left(\frac{2}{1+x}\right)\right).$$

Observe that $\Delta_{f}\left(P||Q\right)=\Delta_{f}\left(Q||P\right)$, and when f(0)<∞, $\Delta_{f}\left(P||Q\right)\leq \sup_{x\in [0,2]}f\left(x\right)<\infty$ for all P,Q since $\frac{dP}{d(P+Q)/2}$, $\frac{dQ}{d(P+Q)/2}\leq 2$. When f(x)=xlogx, the relative entropy’s skew symmetrization is the Jensen–Shannon divergence. When $f\left(x\right)={\left(x-1\right)}^{2}$ up to a normalization constant the $\chi^{2}$-divergence’s skew symmetrization is the Vincze–Le Cam divergence which we state below for emphasis. The work of Topsøe [11] provides more background on this divergence, where it is referred to as the triangular discrimination.

**Definition** **5.** *When $f\left(t\right)=\frac{{\left(t-1\right)}^{2}}{t+1}$, denote the Vincze–Le Cam divergence by*

$$\Delta \left(P||Q\right):=D_{f}\left(P||Q\right).$$

If one denotes the skew symmetrization of the $\chi^{2}$-divergence by $\Delta_{\chi^{2}}$, one can compute easily from (20) that $\Delta_{\chi^{2}}\left(P||Q\right)=\Delta \left(P||Q\right)/2$. We note that although skewing preserves 0-convexity, by the above example, it does not preserve κ-convexity in general. The skew symmetrization of the $\chi^{2}$-divergence a 2-convex divergence while $f\left(t\right)={\left(t-1\right)}^{2}/\left(t+1\right)$ corresponding to the Vincze–Le Cam divergence satisfies $f^{\prime \prime }\left(t\right)=\frac{8}{{\left(t+1\right)}^{3}}$, which cannot be bounded away from zero on (0,∞).

**Corollary** **2.** *For an f-divergence such that f is a κ-convex on (0,2),*

(21) $$\Delta_{f}\left(P||Q\right)\geq \frac{\kappa }{4}\Delta \left(P||Q\right)=\frac{\kappa }{2}\Delta_{\chi^{2}}\left(P||Q\right),$$

*with equality when the $f\left(t\right)={\left(t-1\right)}^{2}$ corresponding the the $\chi^{2}$-divergence, where $\Delta_{f}$ denotes the skew symmetrized divergence associated to f and Δ is the Vincze- Le Cam divergence.*

**Proof.**  Applying Proposition 2

$$\begin{array}{cc} 0 & =D_{f}\left(\frac{P+Q}{2}||\frac{Q+P}{2}\right)\\ \; & \leq \frac{1}{2}D_{f}\left(P||\frac{Q+P}{2}\right)+\frac{1}{2}D_{f}\left(Q||\frac{Q+P}{2}\right)-\frac{\kappa }{8}\int {\left(\frac{2P}{P+Q}-\frac{2Q}{P+Q}\right)}^{2}d\left(P+Q\right)/2\\ \; & =\Delta_{f}\left(P||Q\right)-\frac{\kappa }{4}\;\Delta \left(P||Q\right). \end{array}$$

 □

When f(x)=xlogx, we have $f^{\prime \prime }\left(x\right)\geq \frac{\log e}{2}$ on [0,2], which demonstrates that up to a constant $\frac{\log e}{8}$ the Jensen–Shannon divergence bounds the Vincze–Le Cam divergence (see [11] for improvement of the inequality in the case of the Jensen–Shannon divergence, called the “capacitory discrimination” in the reference, by a factor of 2).

We now investigate more general, non-symmetric skewing in what follows.

**Proposition** **4.** *For α,β∈[0,1], define*

(22) $$C\left(\alpha \right):=\left\{\begin{array}{cc} 1-\alpha & when\;\alpha \leq \beta \\ \alpha & when\;\alpha >\beta , \end{array}\right.$$

*and*

(23) $$S_{\alpha ,\beta }\left(P||Q\right):=D\left(\left(1-\alpha \right)P+\alpha Q||\left(1-\beta \right)P+\beta Q\right).$$

*Then,*

(24) $$S_{\alpha ,\beta }\left(P||Q\right)\leq C\left(\alpha \right)D_{\infty }{\left(\alpha ||\beta )|P-Q\right|}_{TV},$$

*where $D_{\infty }\left(\alpha ||\beta \right):=\log\left(\max\left\{\frac{\alpha }{\beta },\frac{1-\alpha }{1-\beta }\right\}\right)$ is the binary ∞-Rényi divergence [27].*

We need the following lemma originally proved by Audenart in the quantum setting [28]. It is based on a differential relationship between the skew divergence [12] and the [15] (see [29,30]).

**Lemma** **3** (Theorem III.1 [26])**.** *For P and Q probability measures and t∈[0,1],*

(25) $$S_{0,t}\left(P||Q\right)\leq {-\log t|P-Q|}_{TV}.$$

**Proof** **of Theorem 4.** If α≤β, then $D_{\infty }\left(\alpha ||\beta \right)=\log\frac{1-\alpha }{1-\beta }$ and C(α)=1−α. In addition,

(26) $$\left(1-\beta \right)P+\beta Q=t\left((1-\alpha )P+\alpha Q\right)+\left(1-t\right)Q$$

with $t=\frac{1-\beta }{1-\alpha }$, thus

(27) $$\begin{array}{cc} S_{\alpha ,\beta }\left(P||Q\right) & =S_{0,t}\left(\left(1-\alpha \right)P+\alpha Q||Q\right)\\ \; & \leq {\left(-\log t\right)\;|\left(\left(1-\alpha \right)P+\alpha Q\right)-Q|}_{TV}\\ \; & =C\left(\alpha \right)\;D_{\infty }{\left(\alpha ||\beta )\;|P-Q\right|}_{TV}, \end{array}$$

where the inequality follows from Lemma 3. Following the same argument for α>β, so that C(α)=α, $D_{\infty }\left(\alpha ||\beta \right)=\log\frac{\alpha }{\beta }$, and

(28) $$\left(1-\beta \right)P+\beta Q=t\left((1-\alpha )P+\alpha Q\right)+\left(1-t\right)P$$

for $t=\frac{\beta }{\alpha }$ completes the proof. Indeed,

(29) $$\begin{array}{cc} S_{\alpha ,\beta }\left(P||Q\right) & =S_{0,t}\left(\left(1-\alpha \right)P+\alpha Q||P\right)\\ \; & \leq {-\log t\;|\left(\left(1-\alpha \right)P+\alpha Q\right)-P|}_{TV}\\ \; & =C\left(\alpha \right)\;D_{\infty }{\left(\alpha ||\beta )\;|P-Q\right|}_{TV}. \end{array}$$

 □

We recover the classical bound [11,16] of the Jensen–Shannon divergence by the total variation.

**Corollary** **3.** *For probability measure P and Q,*

(30) $$\mathrm{JSD}\left(P||Q\right)\leq {\log2\;|P-Q|}_{TV}$$

**Proof.**  Since $\mathrm{JSD}\left(P||Q\right)=\frac{1}{2}\;S_{0,\frac{1}{2}}\left(P||Q\right)+\frac{1}{2}\;S_{1,\frac{1}{2}}\left(P||Q\right)$. □

Proposition 4 gives a sharpening of Lemma 1 of Nielsen [17], who proved $S_{\alpha ,\beta }\left(P||Q\right)\leq D_{\infty }\left(\alpha ||\beta \right)$, and used the result to establish the boundedness of a generalization of the Jensen–Shannon Divergence.

**Definition** **6** (Nielsen [17])**.** *For p and q densities with respect to a reference measure μ, $w_{i}>0$, such that $\sum_{i=1}^{n}w_{i}=1$ and $\alpha_{i}\in \left[0,1\right]$, define*

(31) $$JS^{\alpha ,w}\left(p:q\right)=\sum_{i=1}^{n}w_{i}\;D(\left(1-\alpha_{i}\right)p+\alpha_{i}q||\left(1-\bar{\alpha }\right)p+\bar{\alpha }q)$$

*where $\sum_{i=1}^{n}w_{i}\alpha_{i}=\bar{\alpha }$.*

Note that, when n=2, $\alpha_{1}=1$, $\alpha_{2}=0$ and $w_{i}=\frac{1}{2}$, $JS^{\alpha ,w}\left(p:q\right)=\mathrm{JSD}\left(p||q\right)$, the usual Jensen–Shannon divergence. We now demonstrate that Nielsen’s generalized Jensen–Shannon Divergence can be bounded by the total variation distance just as the ordinary Jensen–Shannon Divergence.

**Theorem** **5.** *For p and q densities with respect to a reference measure μ, $w_{i}>0$, such that $\sum_{i=1}^{n}w_{i}=1$ and $\alpha_{i}\in \left(0,1\right)$,*

(32) $$\log e\;{\mathrm{Var}}_{w}{\left(\alpha \right)\;|p-q|}_{TV}^{2}\leq JS^{\alpha ,w}\left(p:q\right)\leq A\;H\left(w\right)\;{\left|p-q\right|}_{TV}$$

*where $H\left(w\right):=-\sum_{i}w_{i}\log w_{i}\geq 0$ and $A=\max_{i}\left|\alpha_{i}-{\bar{\alpha }}_{i}\right|$ with ${\bar{\alpha }}_{i}=\sum_{j\neq i}\frac{w_{j}\alpha_{j}}{1-w_{i}}$.*

Note that, since ${\bar{\alpha }}_{i}$ is the *w* average of the $\alpha_{j}$ terms with $\alpha_{i}$ removed, ${\bar{\alpha }}_{i}\in \left[0,1\right]$ and thus A≤1. We need the following Theorem from Melbourne et al. [26] for the upper bound.

**Theorem** **6** ([26] Theorem 1.1)**.** *For $f_{i}$ densities with respect to a common reference measure γ and $\lambda_{i}>0$ such that $\sum_{i=1}^{n}\lambda_{i}=1$,*

(33) $$h_{\gamma }\left(\sum_{i}\lambda_{i}f_{i}\right)-\sum_{i}\lambda_{i}h_{\gamma }\left(f_{i}\right)\leq TH\left(\lambda \right),$$

*where $h_{\gamma }\left(f_{i}\right):=-\int f_{i}\left(x\right)\log f_{i}\left(x\right)d\gamma \left(x\right)$ and $T=\sup_{i}{\left|f_{i}-{\tilde{f}}_{i}\right|}_{TV}$ with ${\tilde{f}}_{i}=\sum_{j\neq i}\frac{\lambda_{j}}{1-\lambda_{i}}f_{j}$.*

**Proof** **of Theorem 5.** We apply Theorem 6 with $f_{i}=\left(1-\alpha_{i}\right)p+\alpha_{i}q$, $\lambda_{i}=w_{i}$, and noticing that in general

(34) $$h_{\gamma }\left(\sum_{i}\lambda_{i}f_{i}\right)-\sum_{i}\lambda h_{\gamma }\left(f_{i}\right)=\sum_{i}\lambda_{i}D(f_{i}||f),$$

we have

(35) $$\begin{array}{cc} JS^{\alpha ,w}\left(p:q\right) & =\sum_{i=1}^{n}w_{i}D(\left(1-\alpha_{i}\right)p+\alpha_{i}q||\left(1-\bar{\alpha }\right)p+\bar{\alpha }q)\\ \; & \leq TH(w). \end{array}$$

It remains to determine $T=\max_{i}{\left|f_{i}-{\tilde{f}}_{i}\right|}_{TV}$,

(36) $$\begin{array}{cc} {\tilde{f}}_{i}-f_{i} & =\frac{f-f_{i}}{1-\lambda_{i}}\\ \; & =\frac{\left(\left(1-\bar{\alpha }\right)p+\bar{\alpha }q\right)-\left(\left(1-\alpha_{i}\right)p+\alpha_{i}q\right)}{1-w_{i}}\\ \; & =\frac{\left(\alpha_{i}-\bar{\alpha }\right)\left(p-q\right)}{1-w_{i}}\\ \; & =\left(\alpha_{i}-{\bar{\alpha }}_{i}\right)\left(p-q\right). \end{array}$$

Thus, $T=\max_{i}\left(\alpha_{i}-{\bar{\alpha }}_{i}\right){\left|p-q\right|}_{TV}=A{\left|p-q\right|}_{TV},$ and the proof of the upper bound is complete.

To prove the lower bound, we apply Pinsker’s inequality, ${2\log e|P-Q|}_{TV}^{2}\leq D\left(P||Q\right),$

(37) $$\begin{array}{cc} JS^{\alpha ,w}\left(p:q\right) & =\sum_{i=1}^{n}w_{i}D(\left(1-\alpha_{i}\right)p+\alpha_{i}q||\left(1-\bar{\alpha }\right)p+\bar{\alpha }q)\\ \; & \geq \frac{1}{2}\sum_{i=1}^{n}w_{i}2\log e\;{\left|\left(\left(1-\alpha_{i}\right)p+\alpha_{i}q\right)-\left(\left(1-\bar{\alpha }\right)p+\bar{\alpha }q\right)\right|}_{TV}^{2}\\ \; & =\log e\;\sum_{i=1}^{n}w_{i}{\left(\alpha_{i}-\bar{\alpha }\right)}^{2}{\left|p-q\right|}_{TV}^{2}\\ \; & =\log e\;{\mathrm{Var}}_{w}\left(\alpha \right)\;{\left|p-q\right|}_{TV}^{2}. \end{array}$$

 □

**Definition** **7.** *Given an f-divergence, densities p and q with respect to common reference measure, $\alpha \in {\left[0,1\right]}^{n}$ and $w\in {\left(0,1\right)}^{n}$ such that $\sum_{i}w_{i}=1$ define its generalized skew divergence*

(38) $$D_{f}^{\alpha ,w}\left(p:q\right)=\sum_{i=1}^{n}w_{i}D_{f}(\left(1-\alpha_{i}\right)p+\alpha_{i}q||\left(1-\bar{\alpha }\right)p+\bar{\alpha }q).$$

*where $\bar{\alpha }=\sum_{i}w_{i}\alpha_{i}$.*

Note that, by Theorem 4, $D_{f}^{\alpha ,w}$ is an *f*-divergence. The generalized skew divergence of the relative entropy is the generalized Jensen–Shannon divergence $JS^{\alpha ,w}$. We denote the generalized skew divergence of the $\chi^{2}$-divergence from *p* to *q* by

(39) $$\chi_{\alpha ,w}^{2}\left(p:q\right):=\sum_{i}w_{i}\chi^{2}(\left(1-\alpha_{i}\right)p+\alpha_{i}q\left|\right|\left(1-\bar{\alpha }p+\bar{\alpha }q\right)$$

Note that, when n=2 and $\alpha_{1}=0$, $\alpha_{2}=1$ and $w_{i}=\frac{1}{2}$, we recover the skew symmetrized divergence in Definition 4

(40) $$D_{f}^{\left(0,1),(1/2,1/2\right)}\left(p:q\right)=\Delta_{f}\left(p||q\right)$$

The following theorem shows that the usual upper bound for the relative entropy by the $\chi^{2}$-divergence can be reversed up to a factor in the skewed case.

**Theorem** **7.** *For p and q with a common dominating measure μ,*

$$\chi_{\alpha ,w}^{2}\left(p:q\right)\leq N_{\infty }\left(\alpha ,w\right)JS^{\alpha ,w}\left(p:q\right).$$

Writing $N_{\infty }\left(\alpha ,w\right)=\max_{i}\max\left\{\frac{1-\alpha_{i}}{1-\bar{\alpha }},\frac{\alpha_{i}}{\bar{\alpha }}\right\}$. For $\alpha \in {\left[0,1\right]}^{n}$ and $w\in {\left(0,1\right)}^{n}$ such that $\sum_{i}w_{i}=1$, we use the notation $N_{\infty }\left(\alpha ,w\right):=\max_{i}e^{D_{\infty }(\alpha_{i}\left|\right|\bar{\alpha })}$ where $\bar{\alpha }\sum_{i}w_{i}\alpha_{i}$.

**Proof.**  By definition,

$$JS^{\alpha ,w}\left(p:q\right)=\sum_{i=1}^{n}w_{i}D(\left(1-\alpha_{i}\right)p+\alpha_{i}q||\left(1-\bar{\alpha }\right)p+\bar{\alpha }q).$$

Taking $P_{i}$ to be the measure associated to $\left(1-\alpha_{i}\right)p+\alpha_{i}q$ and *Q* given by $\left(1-\bar{\alpha }\right)p+\bar{\alpha }q$, then

(41) $$\frac{dP_{i}}{dQ}=\frac{\left(1-\alpha_{i}\right)p+\alpha_{i}q}{\left(1-\bar{\alpha }\right)p+\bar{\alpha }q}\leq \max\left\{\frac{1-\alpha_{i}}{1-\bar{\alpha }},\frac{\alpha_{i}}{\bar{\alpha }}\right\}=e^{D_{\infty }(\alpha_{i}\left|\right|\bar{\alpha })}\leq N_{\infty }\left(\alpha ,w\right).$$

Since f(x)=xlogx, the convex function associated to the usual KL divergence, satisfies $f^{\prime \prime }\left(x\right)=\frac{1}{x}$, *f* is $e^{-D_{\infty }\left(\alpha \right)}$-convex on $\left[0,\sup_{x,i}\frac{dP_{i}}{dQ}\left(x\right)\right]$, applying Proposition 2, we obtain

(42) $$D\left(\sum_{i}w_{i}P_{i}||Q\right)\leq \sum_{i}w_{i}D(P_{i}\left||Q\right)-\frac{\sum_{i}w_{i}\int_{X}{\left(\frac{dP_{i}}{dQ}-\frac{dP}{dQ}\right)}^{2}dQ}{2N_{\infty }\left(\alpha ,w\right)}.$$

Since $Q=\sum_{i}w_{i}P_{i}$, the left hand side of (42) is zero, while

(43) $$\begin{array}{cc} \sum_{i}w_{i}\int_{X}{\left(\frac{dP_{i}}{dQ}-\frac{dP}{dQ}\right)}^{2}dQ & =\sum_{i}w_{i}\int_{X}{\left(\frac{dP_{i}}{dP}-1\right)}^{2}dP\\ \; & =\sum_{i}w_{i}\chi^{2}(P_{i}\left||P\right)\\ \; & =\chi_{\alpha ,w}^{2}\left(p:q\right). \end{array}$$

Rearranging gives,

(44) $$\frac{\chi_{\alpha ,w}^{2}\left(p:q\right)}{2N_{\infty }\left(\alpha ,w\right)}\leq JS^{\alpha ,w}\left(p:q\right),$$

which is our conclusion. □

## 4. Total Variation Bounds and Bayes Risk

In this section, we derive bounds on the Bayes risk associated to a family of probability measures with a prior distribution λ. Let us state definitions and recall basic relationships. Given probability densities ${\left\{p_{i}\right\}}_{i=1}^{n}$ on a space X with respect a reference measure μ and $\lambda_{i}\geq 0$ such that $\sum_{i=1}^{n}\lambda_{i}=1$, define the Bayes risk,

(45) $$R:=R_{\lambda }\left(p\right):=1-\int_{X}\max_{i}\left\{\lambda_{i}p_{i}\left(x\right)\right\}d\mu \left(x\right)$$

If $\ell \left(x,y\right)=1-\delta_{x}\left(y\right)$, and we define $T:=\left(x\right)\arg\max_{i}\lambda_{i}p_{i}\left(x\right)$ then observe that this definition is consistent with, the usual definition of the Bayes risk associated to the loss function *ℓ*. Below, we consider θ to be a random variable on {1,2,…,n} such that $P\left(\theta =i\right)=\lambda_{i}$, and *x* to be a variable with conditional distribution $P\left(X\in A|\theta =i\right)=\int_{A}p_{i}\left(x\right)d\mu \left(x\right)$. The following result shows that the Bayes risk gives the probability of the categorization error, under an optimal estimator.

**Proposition** **5.** *The Bayes risk satisfies*

$$R=\min_{\hat{\theta }}E\ell \left(\theta ,\hat{\theta }\left(X\right)\right)=E\ell \left(\theta ,T\left(X\right)\right)$$

*where the minimum is defined over $\hat{\theta }:X\to \left\{1,2,\ldots ,n\right\}$.*

**Proof.**  Observe that $R=1-\int_{X}\lambda_{T(x)}p_{T(x)}\left(x\right)d\mu \left(x\right)=E\ell \left(\theta ,T\left(X\right)\right)$. Similarly,

$$\begin{array}{cc} E\ell (\theta ,\hat{\theta }\left(X\right)) & =1-\int_{X}\lambda_{\hat{\theta }\left(x\right)}p_{\hat{\theta }\left(x\right)}\left(x\right)d\mu \left(x\right)\\ \; & \geq 1-\int_{X}\lambda_{T(x)}p_{T(x)}\left(x\right)d\mu \left(x\right)=R, \end{array}$$

which gives our conclusion. □

It is known (see, for example, [9,31]) that the Bayes risk can also be tied directly to the total variation in the following special case, whose proof we include for completeness.

**Proposition** **6.** *When n=2 and $\lambda_{1}=\lambda_{2}=\frac{1}{2}$, the Bayes risk associated to the densities $p_{1}$ and $p_{2}$ satisfies*

(46) $$2R=1-|p_{1}-p_{2}{|}_{TV}$$

**Proof.**  Since $p_{T}=\frac{|p_{1}-p_{2}|+p_{1}+p_{2}}{2}$, integrating gives $\int_{X}p_{T}\left(x\right)d\mu \left(x\right)={\left|p_{1}-p_{2}\right|}_{TV}+1$ from which the equality follows. □

Information theoretic bounds to control the Bayes and minimax risk have an extensive literature (see, for example, [9,32,33,34,35]). Fano’s inequality is the seminal result in this direction, and we direct the reader to a survey of such techniques in statistical estimation (see [36]). What follows can be understood as a sharpening of the work of Guntuboyina [9] under the assumption of a κ-convexity.

The function $T\left(x\right)=\arg\max_{i}\left\{\lambda_{i}p_{i}\left(x\right)\right\}$ induces the following convex decompositions of our densities. The density *q* can be realized as a convex combination of $q_{1}=\frac{\lambda_{T}q}{1-Q}$ where $Q=1-\int \lambda_{T}qd\mu$ and $q_{2}=\frac{(1-\lambda_{T})q}{Q}$,

$$q=\left(1-Q\right)q_{1}+Qq_{2}.$$

If we take $p\sum_{i}\lambda_{i}p_{i}$, then *p* can be decomposed as $\rho_{1}=\frac{\lambda_{T}p_{T}}{1-R}$ and $\rho_{2}=\frac{p-\lambda_{T}p_{T}}{R}$ so that

$$p=\left(1-R\right)\rho_{1}+R\rho_{2}.$$

**Theorem** **8.** *When f is κ-convex, on (a,b) with $a=\inf_{i,x}\frac{p_{i}\left(x\right)}{q(x)}$ and $b=\sup_{i,x}\frac{p_{i}\left(x\right)}{q(x)}$*

$$\sum_{i}\lambda_{i}D_{f}(p_{i}\left||q\right)\geq D_{f}\left(R||Q\right)+\frac{\kappa W}{2}$$

*where*

$$W:=W\left(\lambda_{i},p_{i},q\right):=\frac{{\left(1-R\right)}^{2}}{1-Q}\chi^{2}(\rho_{1}\left|\right|q_{1})+\frac{R^{2}}{Q}\chi^{2}(\rho_{2}\left|\right|q_{2})+W_{0}$$

*for $W_{0}\geq 0$.*

$W_{0}$ can be expressed explicitly as

$$W_{0}=\int \left(1-\lambda_{T}\right)Var_{\lambda_{i}\neq T}\left(\frac{p_{i}}{q}\right)d\mu =\int \sum_{i\neq T}\lambda_{i}\frac{|p_{i}-\sum_{j\neq T}\frac{\lambda_{j}}{1-\lambda_{T}}p_{j}{|}^{2}}{q}d\mu ,$$

where for fixed *x*, we consider the variance $Var_{\lambda_{i}\neq T}\left(\frac{p_{i}}{q}\right)$ to be the variance of a random variable taking values $p_{i}\left(x\right)/q\left(x\right)$ with probability $\lambda_{i}/\left(1-\lambda_{T(x)}\right)$ for i≠T(x). Note this term is a non-zero term only when n>2.

**Proof.**  For a fixed *x*, we apply Lemma 1

(47) $$\begin{array}{cc} \sum_{i}\lambda_{i}f\left(\frac{p_{i}}{q}\right) & =\lambda_{T}f\left(\frac{p_{T}}{q}\right)+\left(1-\lambda_{T}\right)\sum_{i\neq T}\frac{\lambda_{i}}{1-\lambda_{T}}f\left(\frac{p_{i}}{q}\right)\\ \; & \geq \lambda_{T}f\left(\frac{p_{T}}{q}\right)+\left(1-\lambda_{T}\right)\left[f\left(\frac{p-\lambda_{T}p_{T}}{q(1-\lambda_{T})}\right)+\frac{\kappa }{2}{\mathrm{Var}}_{\lambda_{i\neq T}}\left(\frac{p_{i}}{q}\right)\right] \end{array}$$

Integrating,

(48) $$\begin{array}{cc} \sum_{i}\lambda_{i}D_{f}(p_{i}\left||q\right)\geq \int \lambda_{T}f\left(\frac{p_{T}}{q}\right)q & +\int \left(1-\lambda_{T}\right)f\left(\frac{-\lambda_{T}p_{T}+\sum_{i}\lambda_{i}p_{i}}{q(1-\lambda_{T})}\right)q+\frac{\kappa }{2}W_{0}, \end{array}$$

where

(49) $$W_{0}=\int \sum_{i\neq T(x)}\frac{\lambda_{i}}{1-\lambda_{T}\left(x\right)}\frac{|p_{i}-\sum_{j\neq T}\frac{\lambda_{j}}{1-\lambda_{T}}p_{j}{|}^{2}}{q}d\mu .$$

Applying the κ-convexity of *f*,

(50) $$\begin{array}{cc} \int \lambda_{T}f\left(\frac{p_{T}}{q}\right)q & =\left(1-Q\right)\int q_{1}f\left(\frac{p_{T}}{q}\right)\\ \; & \geq \left(1-Q\right)\left(f\left(\frac{\int \lambda_{T}p_{T}}{1-Q}\right)+\frac{\kappa }{2}{\mathrm{Var}}_{q_{1}}\left(\frac{p_{T}}{q}\right)\right)\\ \; & =\left(1-Q\right)f\left(\left(1-R\right)/\left(1-Q\right)\right)+\frac{Q\kappa }{2}W_{1}, \end{array}$$

with

(51) $$\begin{array}{cc} W_{1} & :={\mathrm{Var}}_{q_{1}}\left(\frac{p_{T}}{q}\right)\\ \; & ={\left(\frac{1-R}{1-Q}\right)}^{2}{\mathrm{Var}}_{q_{1}}\left(\frac{\lambda_{T}p_{T}}{\lambda_{T}q}\frac{1-Q}{1-R}\right)\\ \; & ={\left(\frac{1-R}{1-Q}\right)}^{2}{\mathrm{Var}}_{q_{1}}\left(\frac{\rho_{1}}{q_{1}}\right)\\ \; & ={\left(\frac{1-R}{1-Q}\right)}^{2}\chi^{2}(\rho_{1}\left|\right|q_{1}) \end{array}$$

Similarly,

(52) $$\begin{array}{cc} \int \left(1-\lambda_{T}\right)f\left(\frac{p-\lambda_{T}p_{T}}{q(1-\lambda_{T})}\right)q & =Q\int q_{2}f\left(\frac{p-\lambda_{T}p_{T}}{q(1-\lambda_{T})}\right)\\ \; & \geq Qf\left(\int q_{2}\frac{p-\lambda_{T}p_{T}}{q(1-\lambda_{T})}\right)+\frac{Q\kappa }{2}W_{2}\\ \; & =Qf\left(\frac{R}{1-Q}\right)+\frac{Q\kappa }{2}W_{2} \end{array}$$

where

(53) $$\begin{array}{cc} W_{2} & :={\mathrm{Var}}_{q_{2}}\left(\frac{p-\lambda_{T}p_{T}}{q(1-\lambda_{T})}\right)\\ \; & ={\left(\frac{R}{Q}\right)}^{2}{\mathrm{Var}}_{q_{2}}\left(\frac{p-\lambda_{T}p_{T}}{q(1-\lambda_{T})}\frac{Q}{R}\right)\\ \; & ={\left(\frac{R}{Q}\right)}^{2}{\mathrm{Var}}_{q_{2}}{\left(\frac{p-\lambda_{T}p_{T}}{q(1-\lambda_{T})}-\frac{R}{Q}\right)}^{2}\\ \; & ={\left(\frac{R}{Q}\right)}^{2}\int q_{2}{\left(\frac{\rho_{2}}{q_{2}}-1\right)}^{2}\\ \; & ={\left(\frac{R}{Q}\right)}^{2}\chi^{2}(\rho_{2}\left|\right|q_{2}) \end{array}$$

Writing $W=W_{0}+W_{1}+W_{2}$, we have our result. □

**Corollary** **4.** *When $\lambda_{i}=\frac{1}{n}$, and f is κ-convex on $\left(\inf_{i,x}p_{i}/q,\sup_{i,x}p_{i}/q\right)$*

(54) $$\begin{array}{cc} \frac{1}{n}\sum_{i} & D_{f}(p_{i}\left||q\right)\\ \; & \geq D_{f}\left(R||\left(n-1\right)/n\right)+\frac{\kappa }{2}\left(n^{2}{\left(1-R\right)}^{2}\chi^{2}(\rho_{1}\left||q\right)+{\left(\frac{nR}{n-1}\right)}^{2}\chi^{2}(\rho_{2}\left||q\right)+W_{0}\right) \end{array}$$

*further when n=2,*

(55) $$\begin{array}{cc} \frac{D_{f}(p_{1}\left||q\right)+D_{f}(p_{2}\left||q\right)}{2}\geq D_{f} & \left(\frac{1-|p_{1}-p_{2}{|}_{TV}}{2}||\frac{1}{2}\right)\\ \; & +\frac{\kappa }{2}\left(\left(1+\right|p_{1}-p_{2}{|}_{TV}{)}^{2}\chi^{2}(\rho_{1}\left||q)+(1-\right|p_{1}-p_{2}{|}_{TV}{)}^{2}\chi^{2}(\rho_{2}\left||q\right)\right). \end{array}$$

**Proof.**  Note that $q_{1}=q_{2}=q$, since $\lambda_{i}=\frac{1}{n}$ implies $\lambda_{T}=\frac{1}{n}$ as well. In addition, $Q=1-\int \lambda_{T}qd\mu =\frac{n-1}{n}$ so that applying Theorem 8 gives

(56) $$\sum_{i=1}^{n}D_{f}(p_{i}\left||q\right)\geq nD_{f}\left(R||\left(n-1\right)/n\right)+\frac{\kappa nW(\lambda_{i},p_{i},q)}{2}.$$

The term *W* can be simplified as well. In the notation of the proof of Theorem 8,

(57) $$\begin{array}{cc} W_{1} & =n^{2}{\left(1-R\right)}^{2}\chi^{2}\left(\rho_{1},q\right)\\ W_{2} & ={\left(\frac{nR}{n-1}\right)}^{2}\chi^{2}(\rho_{2}\left||q\right)\\ W_{0} & =\int \frac{\frac{1}{n-1}\sum_{i\neq T}{\left(p_{i}-\frac{1}{n-1}\sum_{j\neq T}p_{j}\right)}^{2}}{q}d\mu . \end{array}$$

For the special case, one needs only to recall $R=\frac{1-|p_{1}-p_{2}{|}_{TV}}{2}$ while inserting 2 for *n*. □

**Corollary** **5.** *When $p_{i}\leq q/t^{*}$ for $t^{*}>0$, and f(x)=xlogx*

$$\sum_{i}\lambda_{i}D(p_{i}\left||q\right)\geq D\left(R||Q\right)+\frac{t^{*}W\left(\lambda_{i},p_{i},q\right)}{2}$$

*for $D(p_{i}||q)$ the relative entropy. In particular,*

$$\sum_{i}\lambda_{i}D(p_{i}\left||q\right)\geq D\left(p||q\right)+D\left(R||P\right)+\frac{t^{*}W\left(\lambda_{i},p_{i},p\right)}{2}$$

*where $P=1-\int \lambda_{T}pd\mu$ for $p=\sum_{i}\lambda_{i}p_{i}$ and $t^{*}=\min\lambda_{i}$.*

**Proof.**  For the relative entropy, f(x)=xlogx is $\frac{1}{M}$-convex on [0,M] since $f^{\prime \prime }\left(x\right)=1/x$. When $p_{i}\leq q/t^{*}$ holds for all *i*, then we can apply Theorem 8 with $M=\frac{1}{t^{*}}$. For the second inequality, recall the compensation identity, $\sum_{i}\lambda_{i}D(p_{i}\left||q\right)=\sum_{i}\lambda_{i}D(p_{i}\left||p\right)+D\left(p||q\right)$, and apply the first inequality to $\sum_{i}D(p_{i}\left||p\right)$ for the result.  □

This gives an upper bound on the Jensen–Shannon divergence, defined as $\mathrm{JSD}\left(\mu ||\nu \right)=\frac{1}{2}D\left(\mu ||\mu /2+\nu /2\right)+\frac{1}{2}D\left(\nu ||\mu /2+\nu /2\right)$. Let us also note that through the compensation identity $\sum_{i}\lambda_{i}D(p_{i}\left||q\right)=\sum_{i}\lambda_{i}D(p_{i}\left||p\right)+D\left(p||q\right)$, $\sum_{i}\lambda_{i}D(p_{i}\left||q\right)\geq \sum_{i}\lambda_{i}D(p_{i}\left||p\right)$ where $p=\sum_{i}\lambda_{i}p_{i}$. In the case that $\lambda_{i}=\frac{1}{N}$

(58) $$\begin{array}{cc} \sum_{i}\lambda_{i} & D(p_{i}||q)\\ \; & \geq \sum_{i}\lambda_{i}D(p_{i}\left||p\right)\\ \; & \geq Qf\left(\frac{1-R}{Q}\right)+\left(1-Q\right)f\left(\frac{R}{1-Q}\right)+\frac{t^{*}W}{2} \end{array}$$

**Corollary** **6.** *For two densities $p_{1}$ and $p_{2}$, the Jensen–Shannon divergence satisfies the following,*

(59) $$\begin{array}{cc} \mathrm{JSD}(p_{1}||p_{2})\geq D & \left(\frac{1-|p_{1}-p_{2}{|}_{TV}}{2}||1/2\right)\\ \; & +\frac{1}{4}\left(\left(1+\right|p_{1}-p_{2}{|}_{TV}{)}^{2}\chi^{2}(\rho_{1}\left||p)+(1-\right|p_{1}-p_{2}{|}_{TV}{)}^{2}\chi^{2}(\rho_{2}\left||p\right)\right) \end{array}$$

*with ρ(i) defined above and $p=p_{1}/2+p_{2}/2$.*

**Proof.**  Since $\frac{p_{i}}{(p_{1}+p_{2})/2}\leq 2$ and f(x)=xlogx satisfies $f^{\prime \prime }\left(x\right)\geq \frac{1}{2}$ on (0,2). Taking $q=\frac{p_{1}+p_{2}}{2}$, in the n=2 example of Corollary 4 with $\kappa =\frac{1}{2}$ yields the result. □

Note that $2D\left(\left(1+V\right)/2||1/2\right)=\left(1+V\right)\log\left(1+V\right)+\left(1-V\right)\log\left(1-V\right)\geq V^{2}\log e$, we see that a further bound,

(60) $$\mathrm{JSD}(p_{1}\left|\right|p_{2})\geq \frac{\log e}{2}V^{2}+\frac{{\left(1+V\right)}^{2}\chi^{2}(\rho_{1}\left||p\right)+{\left(1-V\right)}^{2}\chi^{2}(\rho_{2}\left||p\right)}{4},$$

can be obtained for $V=|p_{1}-p_{2}{|}_{TV}$.

### On Topsøe’s Sharpening of Pinsker’s Inequality

For $P_{i},Q$ probability measures with densities $p_{i}$ and *q* with respect to a common reference measure, $\sum_{i=1}^{n}t_{i}=1$, with $t_{i}>0$, denote $P=\sum_{i}t_{i}P_{i}$, with density $p=\sum_{i}t_{i}p_{i}$, the compensation identity is

(61) $$\sum_{i=1}^{n}t_{i}D(P_{i}\left||Q\right)=D\left(P||Q\right)+\sum_{i=1}^{n}t_{i}D(P_{i}||P).$$

**Theorem** **9.** *For $P_{1}$ and $P_{2}$, denote $M_{k}=2^{-k}P_{1}+\left(1-2^{-k}\right)P_{2}$, and define*

$$\begin{array}{c} M_{1}\left(k\right)=\frac{M_{k}1_{\left\{P_{1}>P_{2}\right\}}+P_{2}1_{\left\{P_{1}\leq P_{2}\right\}}}{M_{k}\left\{P_{1}>P_{2}\right\}+P_{2}\left\{P_{1}\leq P_{2}\right\}}\;M_{2}\left(k\right)=\frac{M_{k}1_{\left\{P_{1}\leq P_{2}\right\}}+P_{2}1_{\left\{P_{1}>P_{2}\right\}}}{M_{k}\left\{P_{1}\leq P_{2}\right\}+P_{2}\left\{P_{1}>P_{2}\right\}}, \end{array}$$

*then the following sharpening of Pinsker’s inequality can be derived,*

$$\begin{array}{c} D(P_{1}\left|\right|P_{2})\geq \left(2\log e\right)|P_{1}-P_{2}{|}_{TV}^{2}+\sum_{k=0}^{\infty }2^{k}\left(\frac{\chi^{2}\left(M_{1}\left(k\right),M_{k+1}\right)}{2}+\frac{\chi^{2}\left(M_{2}\left(k\right),M_{k+1}\right)}{2}\right). \end{array}$$

**Proof.**  When n=2 and $t_{1}=t_{2}=\frac{1}{2}$, if we denote $M=\frac{P_{1}+P_{2}}{2}$, then (61) reads as

(62) $$\frac{1}{2}D(P_{1}\left||Q\right)+\frac{1}{2}D(P_{2}\left||Q\right)=D(M||Q)+\mathrm{JSD}(P_{1}\left|\right|P_{2}).$$

Taking $Q=P_{2}$, we arrive at

(63) $$D(P_{1}\left|\right|P_{2})=2D(M||P_{2})+2\mathrm{JSD}(P_{1}\left|\right|P_{2})$$

Iterating and writing $M_{k}=2^{-k}P_{1}+\left(1-2^{-k}\right)P_{2}$, we have

(64) $$D(P_{1}\left|\right|P_{2})=2^{n}\left(D(M_{n}\left|\right|P_{2})+2\sum_{k=0}^{n}\mathrm{JSD}(M_{n}\left|\right|P_{2})\right)$$

It can be shown (see [11]) that $2^{n}D(M_{n}\left|\right|P_{2})\to 0$ with n→∞, giving the following series representation,

(65) $$D(P_{1}\left|\right|P_{2})=2\sum_{k=0}^{\infty }2^{k}\mathrm{JSD}(M_{k}\left|\right|P_{2}).$$

Note that the ρ-decomposition of $M_{k}$ is exactly $\rho_{i}=M_{k}\left(i\right)$, thus, by Corollary 6,

(66) $$\begin{array}{cc} D(P_{1}||P_{2}) & =2\sum_{k=0}^{\infty }2^{k}\mathrm{JSD}(M_{k}\left|\right|P_{2})\\ \; & \geq \sum_{k=0}^{\infty }2^{k}\left(|M_{k}-P_{2}{|}_{TV}^{2}\log e+\frac{\chi^{2}\left(M_{1}\left(k\right),M_{k+1}\right)}{2}+\frac{\chi^{2}\left(M_{2}\left(k\right),M_{k+1}\right)}{2}\right)\\ \; & =\left(2\log e\right)|P_{1}-P_{2}{|}_{TV}^{2}+\sum_{k=0}^{\infty }2^{k}\left(\frac{\chi^{2}\left(M_{1}\left(k\right),M_{k+1}\right)}{2}+\frac{\chi^{2}\left(M_{2}\left(k\right),M_{k+1}\right)}{2}\right). \end{array}$$

Thus, we arrive at the desired sharpening of Pinsker’s inequality. □

Observe that the k=0 term in the above series is equivalent to

(67) $$2^{0}\left(\frac{\chi^{2}\left(M_{1}\left(0\right),M_{0+1}\right)}{2}+\frac{\chi^{2}\left(M_{2}\left(0\right),M_{0+1}\right)}{2}\right)=\frac{\chi^{2}\left(\rho_{1},p\right)}{2}+\frac{\chi^{2}\left(\rho_{2},p\right)}{2},$$

where $\rho_{i}$ is the convex decomposition of $p=\frac{p_{1}+p_{2}}{2}$ in terms of $T\left(x\right)=\arg\max\{p_{1}\left(x\right),p_{2}\left(x\right)\}$.

## 5. Conclusions

In this article, we begin a systematic study of strongly convex divergences, and how the strength of convexity of a divergence generator *f*, quantified by the parameter κ, influences the behavior of the divergence $D_{f}$. We prove that every strongly convex divergence dominates the square of the total variation, extending the classical bound provided by the $\chi^{2}$-divergence. We also study a general notion of a skew divergence, providing new bounds, in particular for the generalized skew divergence of Nielsen. Finally, we show how κ-convexity can be leveraged to yield improvements of Bayes risk *f*-divergence inequalities, and as a consequence achieve a sharpening of Pinsker’s inequality.

## Figures and Tables

**Table 1:** Examples of Strongly Convex Divergences.

Divergence	*f*	κ	Domain
relative entropy (KL)	tlogt	$\frac{1}{M}$	(0,M]
total variation	$\frac{\left|t-1\right|}{2}$	0	(0,∞)
Pearson’s $\chi^{2}$	${\left(t-1\right)}^{2}$	2	(0,∞)
squared Hellinger	$2(1-\sqrt{t})$	$M^{-\frac{3}{2}}/2$	(0,M]
reverse relative entropy	−logt	$1/M^{2}$	(0,M]
Vincze- Le Cam	$\frac{{\left(t-1\right)}^{2}}{t+1}$	$\frac{8}{{\left(M+1\right)}^{3}}$	(0,M]
Jensen–Shannon	$\left(t+1\right)\log\frac{2}{t+1}+t\log t$	$\frac{1}{M(M+1)}$	(0,M]
Neyman’s $\chi^{2}$	$\frac{1}{t}-1$	$2/M^{3}$	(0,M]
Sason’s *s*	$\log{\left(s+t\right)}^{{\left(s+t\right)}^{2}}-\log{\left(s+1\right)}^{{\left(s+1\right)}^{2}}$	2log(s+M)+3	[M,∞), $s>e^{-3/2}$
α-divergence	$\frac{4\left(1-t^{\frac{1+\alpha }{2}}\right)}{1-\alpha^{2}},\;\alpha \neq \pm 1$	$M^{\frac{\alpha -3}{2}}$	$\left\{\begin{array}{c} {}[M,\infty ),\;\alpha >3\\ (0,M],\;\alpha <3 \end{array}\right.$

## Appendix A

**Theorem** **A1.**
*The class of f-divergences is stable under skewing. That is, if f is convex, satisfying f(1)=0, then*

(A1) $$\begin{array}{c} \hat{f}\left(x\right):=\left(tx+\left(1-t\right)\right)f\left(\frac{rx+(1-r)}{tx+(1-t)}\right) \end{array}$$

*is convex with $\hat{f}\left(1\right)=0$ as well.*

**Proof.** If μ and ν have respective densities *u* and *v* with respect to a reference measure γ, then rμ+(1−r)ν and tμ+1−tν have densities ru+(1−r)v and tu+(1−t)v

(A2) $$\begin{array}{cc} S_{f,r,t}\left(\mu ||\nu \right) & =\int f\left(\frac{ru+(1-r)v}{tu+(1-t)v}\right)\left(tu+\left(1-t\right)v\right)d\gamma \end{array}$$

(A3) $$\begin{array}{cc} \; & =\int f\left(\frac{r\frac{u}{v}+\left(1-r\right)}{t\frac{u}{v}+\left(1-t\right)}\right)\left(t\frac{u}{v}+\left(1-t\right)\right)vd\gamma \end{array}$$

(A4) $$\begin{array}{cc} \; & =\int \hat{f}\left(\frac{u}{v}\right)vd\gamma . \end{array}$$

Since $\hat{f}\left(1\right)=f\left(1\right)=0$, we need only prove $\hat{f}$ convex. For this, recall that the conic transform *g* of a convex function *f* defined by g(x,y)=yf(x/y) for y>0 is convex, since

(A5) $$\begin{array}{cc} \frac{y_{1}+y_{2}}{2}f\left(\frac{x_{1}+x_{2}}{2}/\frac{y_{1}+y_{2}}{2}\right)\; & =\frac{y_{1}+y_{2}}{2}f\left(\frac{y_{1}}{y_{1}+y_{2}}\frac{x_{1}}{y_{1}}+\frac{y_{2}}{y_{1}+y_{2}}\frac{x_{2}}{y_{2}}\right) \end{array}$$

(A6) $$\begin{array}{cc} \; & \leq \frac{y_{1}}{2}f\left(x_{1}/y_{1}\right)+\frac{y_{2}}{2}f\left(x_{2}/y_{2}\right). \end{array}$$

Our result follows since $\hat{f}$ is the composition of the affine function A(x)=(rx+(1−r),tx+(1−t)) with the conic transform of *f*,

(A7) $$\begin{array}{c} \hat{f}\left(x\right)=g\left(A\left(x\right)\right). \end{array}$$

 □
